# Do Criminal Offenders Have a Right to Neurorehabilitation?

**DOI:** 10.1007/s11572-022-09630-y

**Published:** 2022-03-29

**Authors:** Emma Dore-Horgan

**Affiliations:** University of Oxford (Oxford Uehiro Centre and Faculty of Philosophy), Oxford, UK

**Keywords:** Neurointerventions, Right to rehabilitation, Offender rights, Right to hope, Penal policy

## Abstract

Soon it may be possible to promote the rehabilitation of criminal offenders through *neurointerventions* (interventions which exert direct physical, chemical or biological effects on the brain). Some jurisdictions already utilise neurointerventions to diminish the risk of sexual or drug-related reoffending. And investigation is underway into several other neurointerventions that might also have rehabilitative applications within criminal justice—for example, pharmacotherapy to reduce aggression or impulsivity. Ethical debate on the use of neurointerventions to facilitate rehabilitation—henceforth ‘neurorehabilitation’—has proceeded on two assumptions: that we have instrumental reasons for employing neurorehabilitation (e.g. because it helps protect the public from crime); and that its permissibility depends upon whether its use unjustifiably infringes offenders’ rights. This paper defends a different, hitherto neglected thought. I argue we have rights-based reasons to offer neurorehabilitation to offenders—in other words, that offenders have a *moral right* to neurorehabilitation. I identify three considerations which support a moral right to conventional rehabilitative interventions—(1) as a countermeasure to the debilitating side-effects of punishment; (2) as a derivative right of the right to hope for renewed liberty; and (3) as compensation for structural injustice. I argue these considerations extend to support a moral right to neurorehabilitation in the following instance: when neurorehabilitation would be part of the *most effective* package for facilitating rehabilitation, and can be carried out at reasonable cost. I then defend my argument against potential objections, including the objection that neurorehabilitation is a bad option for offenders to have and the charge of over-medicalisation.

## Introduction

There is reason to believe that, in the future, it may be possible to promote the rehabilitation of criminal offenders^1^ through *neurointerventions* (interventions which exert direct physical, chemical or biological effects on the brain).^2^ Some jurisdictions already utilise neurointerventions to diminish the risk of sexual or drug-related reoffending (using anti-libidinal medications in the case of the former kinds of offences^3^ and opioid substitution therapy in the latter).^4^ And investigation is underway into several other neurointerventions that might also have anti-recidivist or broader rehabilitative applications within criminal justice—for example, pharmacological and/or brain stimulation techniques to reduce impulsivity or to boost empathy.^5^ Ethical debate on the use of neurointerventions to facilitate rehabilitation within criminal justice systems—henceforth called ‘neurorehabilitation’—has proceeded on two assumptions: that we have instrumental reasons to employ neurorehabilitation (e.g. because it helps to protect the public from crime); and that the permissibility of these interventions depends upon whether their use unjustifiably infringes offender rights.^6^ In this paper, however, I explore and defend a different thought, one that has not been voiced in the literature to date. I argue we have rights-based reasons to offer neurorehabilitation to offenders. In other words, that offenders have a *moral right* to neurorehabilitation. My contention is that arguments supporting a moral right to conventional rehabilitative interventions extend to support a moral right to neurorehabilitation in the following instance: when neurorehabilitation would be part of the *most effective* package for facilitating rehabilitation, and can be carried out safely and at reasonable cost.

Some clarifications. When speaking of a right to rehabilitation and neurorehabilitation, I mean a right to *types of interventions* that seek to bring about dispositional changes in offenders such that they are no longer inclined to engage in crime. Examples might include behavioural therapies, educational initiatives or (in the case of *neuro*rehabilitation) pharmaceuticals aimed at attenuating aggression, improving impulse-control or enhancing empathic abilities. The term ‘rehabilitation’ is used to refer both to these kinds of interventions and to the dispositional changes they are intended to produce (and I will use the term in reference to each of these meanings throughout). But when speaking of a *right*, I am only referring to a right to the abovementioned kinds of interventions, not to a right to the dispositional change. I am also not including, within the classes of rehabilitation and neurorehabilitation, interventions which aim to merely disincentivise crime, condition against it or make it physically impossible. Measures which seek to simply condition offenders to refrain from crime by pairing socially undesirable thoughts with an aversive stimulus, for example, thus do not count as (neuro)rehabilitation on my understanding.^7^ I assume also that we will have at our disposal, in the medium-term future, a number of neurorehabilitative options that are safe and reasonably affordable to employ. This is by no means an unreasonable assumption, given that some candidate neurorehabilitative tools are currently approved as safe for use in other contexts;^8^ and effective neurorehabilitation, even if expensive, would plausibly compare favourably to the already costly practice of continued or repeat incarceration.^9^ Finally, this paper is only addressing the question of whether offenders have a *right to be offered* neurorehabilitation. It is not exploring whether offenders have a duty to neurorehabilitate themselves or whether the State has a right to impose neurorehabilitation.

### Current Failure to Recognise a Moral Right to Neurorehabilitation

The emerging legal picture in Europe and the United States is supportive of offenders’ moral right to rehabilitation more generally. The former’s Court of Human Rights (ECtHR) has indicated rehabilitation is a ‘positive obligation’ of States^10^ and offenders must be provided with ‘realistic opportunity’ to rehabilitate themselves.^11^ German case law acknowledges ‘penal institutions [are obliged] … to promote the rehabilitation of the inmates’.^12^ And international human rights instruments similarly require that States provide rehabilitation, with Article 10(3) of the International Covenant on Civil and Political Rights (ICCPR) stipulating that ‘penitentiary system(s) shall comprise treatment of prisoners the essential aim of which shall be their reformation and social rehabilitation’.^13^

A number of moral arguments have, moreover, been advanced in support of this moral right to rehabilitation and its legal recognition. Three, in particular, are persuasive. A first holds that offenders have such a right because they are entitled to have any debilitating side-effects of State punishment offset. While offenders might be liable to *punishment*, so the reasoning goes, they are not liable to deterioration in functioning that extends beyond their sentences. They thus have a right not to finish their sentences even less capable of pursuing a crime-free lifestyle and with even lower functioning than they had at the outset, and rehabilitation is presented as one way of forestalling these outcomes. A second justification views rehabilitation as a derivative right of the right to genuine hope for renewed liberty; or perhaps, more generally, the right to hope for the chance to turn one’s life around in the future. And a third (somewhat less prominent) argument grounds a right to rehabilitation in offenders’ entitlement to have structural injustices—specifically, injustices which contributed to their coming into contact with criminal justice institutions—redressed by these very institutions.^14^ Rehabilitation is here again viewed as one concrete way of providing such recompense.

Yet, despite this emerging recognition of offenders’ right to rehabilitation, there has been no discussion as to whether this right implies a right to *neuro*rehabilitation. Indeed, efforts to defend a moral right to neurorehabilitation are conspicuously absent from both the literature on criminal rehabilitation and the scholarly debate surrounding neurointervention use within criminal justice. Some protagonists in the latter debate have argued offenders have rights *against* neurorehabilitation—rights not to be subjected to neurorehabilitation against their will.^15^ But the question of whether they have a right *to* it remains unaddressed.

Case law defending offenders’ entitlement to rehabilitation, moreover, exclusively focuses on conventional, non-biological forms of rehabilitation (e.g. educational interventions, counselling, behavioural therapy and social interventions) as the relevant interventions at issue when it comes to satisfying this right.^16^ Neurointerventions and other biological approaches only receive mention in the context of treating recognised mental health disorders or diseases;^17^ and some authors have suggested neurorehabilitation ought *only* be provided when it is coextensive with the treatment of ‘physical or mental illnesses’ and not for the alleviation of criminogenic risk factors.^18^ Moreover, even when neurorehabilitation *would* be coextensive with the treatment of recognised diseases or disorders (as in the case of pharmacotherapy to address substance abuse), offenders in some jurisdictions cannot always access available neurointerventions. And this is even in jurisdictions where a State obligation to rehabilitate is recognised and conventional forms of rehabilitation *are* being offered. For example, recent research indicates that most prisoners with opioid use disorder *do not* receive opioid substitution therapy (OST) within correctional settings in the United States (despite much evidence to support its efficacy),^19^ with rehabilitative efforts very much focused on drug education and behavioural counselling rather than a more multimodal rehabilitative package.^20^ There is thus reason to believe that, if offenders *do* have a right to neurorehabilitation, it is a right that is frequently infringed—perhaps more frequently than the general right to rehabilitation.

The above observations suggest we are currently neglecting to include a whole class of interventions within the purview of offenders’ entitlements. This neglect is something this paper hopes to remedy.

The article’s structure is as follows. Sections 2, 3 and 4 outline the abovementioned oft-cited grounds of a moral right to rehabilitation more generally: (1) as a countermeasure to the debilitating side-effects of punishment; (2) as a derivative right of the right to justified hope for renewed liberty; and (3) as compensation for structural injustice. I maintain, in each section, that these considerations are persuasive, argue they *also* support a moral right to neurorehabilitation and defend this line of argument against some preliminary objections. Section 5 anticipates and addresses two further challenges: the objection that neurorehabilitation is a bad option for offenders to have and the charge of over-medicalisation. Section 6 offers some concluding comments.

## The Non-Degeneration Justification

A first justification for a right to rehabilitation, notably voiced by Rotman,^21^ springs from the fact that we punish offenders—or more specifically, from the fact that our punishment practices have the potential to be (and often are) debilitating for offenders. The degenerative effects of imprisonment (the dominant method of punishment used today for serious offences) are well-documented.^22^ Imprisonment can precipitate a decline in people’s ability to interact socially.^23^ It can compromise people’s capacity to make decisions for themselves,^24^ can have a brutalising influence,^25^ and can bring about a decline in brain functions connected to self-regulation.^26^ Additionally, and most obviously, imprisonment can be traumatising for those subjected to it, precipitating an increase in negative emotions and a deterioration in mental health—something which might, in turn (though the evidence on this is mixed), place these individuals at a higher risk of recidivism and repeat incarceration.^27^

Of course, the specific conditions of the imposed prison regime undoubtedly make a difference. And non-carceral punishments are unlikely to give rise to all, or the same level of, these debilitating effects. But the point is that one prominent mode of State punishment, regardless of the imposed regime or security level, has the potential to give rise to at least *some* of these effects because of the restrictions and deprivations it necessarily involves. And while there may be several alternative punishments we could (and do) utilise which are less debilitating, many alternatives to incarceration—such as probation or house arrest—also risk producing at least some of these negative outcomes.^28^ It is this very fact that serves as one ground for a right to rehabilitation: the right to be provided with rehabilitation as a means to counteract the debilitating side-effects of punishment.

This first justification is a minimal and negative one. The argument is not that offenders have a right to opportunities that might enhance their chances of leading a law-abiding and/or flourishing life beyond that which they had before committing crime. Rather, the claim is offenders have a right to avoid having their chances of refraining from crime *additionally* diminished through punishment. The underlying rationale for this claim (offered in *Laaman v. Helgemoe* and elsewhere) is that degenerative criminal justice experiences would ‘do violence to [offenders’] intrinsic worth and dignity’, contravening their rights against cruel and/or disproportionate punishment.^29^ And the argument is that, given rehabilitation is one salient way to counteract punishment’s detrimental side-effects, a right to rehabilitation is one derivative right of this more general entitlement not to degenerate through punishment.

We can readily see how this entitlement justifies the provision of many conventional forms of rehabilitation such as social skills training, counselling, mindfulness-based interventions and enrichment activities. I argue here, however, that this line of reasoning also supports a right to *neuro*rehabilitation in situations where affordable neurorehabilitation would counter the unwarranted harms of punishment more effectively than conventional rehabilitation would do alone. In arguing for this, I am assuming that the right to have the unwarranted harms of punishment offset implies a right to the most effective package of interventions for neutralising these harms from among the range of affordable options. Such a view seems to be accepted when it comes to offering traditional forms of rehabilitation: that while offenders do not have a right to *all and every* rehabilitative intervention, they do have a right to the most effective interventions from among the reasonably affordable alternatives. My contention here, however, is that the most effective approach will—in the medium-term future—often involve neurorehabilitation.

I anticipate the reply that offenders would be *better* served if we were to transform our punishment practices rather than merely seeking novel routes to neutralise their debilitating side-effects. I also anticipate that some will doubt that neurorehabilitation will often be part of the most effective package for countering punishment’s degenerative impact. Surely, they might ask, the provision of a *sufficient* level of environmental and psychosocial supports could be just as, if not more, effective—so why think the right to the most effective approach might often imply a right to neurorehabilitation?

In response, let me say the following. Firstly, I concur with the view that we ought to ameliorate our present punishment practices such that the issue of offender degeneration does not arise (or at least has very low prevalence). I agree also that this should take priority. However, for as long as we *do* utilise punishments which run the risk of offender degeneration—and it is plausible that we will always have to rely on some sort of detention-based sanctions in the case of offenders who pose a serious risk to the public—then a right to the countering force of rehabilitation applies. And I maintain that this right includes a right to neurorehabilitation, if and when such intervention can be expected to be part of the most effective, affordable protection against such a decline.

The question of whether neurorehabilitation *will* in the future be part of the most effective approach is, of course, an empirical one. However, it is reasonable to postulate that it might be, and thus reasonable to see the above line of argument as having practical significance. Much neuroscientific work on psychological resilience, for instance, indicates that certain neurobiological states are associated with greater resilience in individuals—psychological resilience being the ability to adapt well and maintain a normal or near-normal level of functioning in the face of adversity and stress.^30^ Promising research is also currently being carried out on how to promote these biological mechanisms of resilience through pharmacotherapy, with a view to harnessing such drugs in the management of depression and post-traumatic stress.^31^ And such drugs may, in the not too distant future, also have important application when it comes to prison populations - particularly since evidence suggests that conventional methods of fostering resilience may be *less effective* and/or require a *longer timeframe* to achieve results when individuals have already been exposed to trauma,^32^ as many prisoners indubitably have.^33^ Clearly then, *additional, faster-acting* approaches to resilience-building would be an important component of the most effective package for countering prisoner degeneration in many instances. This gives us at least one concrete example where a right to the most effective measures against degeneration could soon imply a right to neurorehabilitation in the form of resilience-enhancing pharmacotherapy.

The critic might protest at this juncture that a right against degeneration does not entail a right to *passively* have one’s resilience, or any other relevant antidotes to degeneration, boosted in the face of adversity. To continue with the example of resilience, the contention could be that offenders are only entitled to services to help them cultivate resilience *for themselves* and *through their own efforts*, and not to interventions which directly enhance biological mechanisms of resilience. And the reasoning for this might be that direct enhancement of resilience through neurointervention would hinder the realisation of the aims of punishment. In other words, that directly enabling offenders to ‘bounce back’ when faced with punishment’s burdens would undermine the censorious message punishment aims to impart or would prevent offenders from experiencing the hard treatment retributivists argue they deserve.

In response to these protests, I offer two replies. Firstly, while a right to rehabilitation denotes a right to avail of rehabilitative interventions rather than to the achievement of a rehabilitated state, a right against degeneration is precisely the right to *not degenerate* through punishment. There is consequently no prerequisite that offenders have to ‘work’ to combat the degenerative impact punishment might have on them. Non-degeneration is something the State should strive to make possible. And while many potential antidotes to degeneration inevitably *will* require offender input and effort, it is not the case that offenders are *only* entitled to those measures requiring effort on their part.

Secondly, I dispute the suggestion that neurorehabilitation to counter degeneration would thwart the realisation of punishment’s goals. In targeting the problem of degeneration, the aim is to combat punishment’s *unwanted and unwarranted side-effects*. The intended burdens of punishment would still persist. An offender sentenced to a period of imprisonment would still have their liberty restricted, their autonomy curtailed and their privacy and freedom of association seriously circumscribed. Another offender, under supervision within the community, would still face the burdens of restricted autonomy and time, and the pains of being forced to return to discussing the offence and/or the victim at probation meetings.^34^ Enhancing resilience in the face of such burdens is not to cancel out their burdensome impact. Offenders are not being provided with ‘happy pills’ (whatever these might be) such that they are oblivious to the harms of punishment. Instead, efforts are merely being taken to facilitate offender’s coping abilities such that they do not experience *additional* (foreseeable) burdens. And it is hard to see how this would be inconsistent with the realisation of punishment’s goals so long as the intended burdens of the sanction remain proportionate.

There is, of course, a further concern (hinted at already) that neurorehabilitation to combat offender degeneration would divert attention from the need to improve our current carceral practices. In other words, that States would be less inclined to reform criminal justice institutions if they recognised that offenders’ rights against non-degeneration could be fulfilled through the delivery of neurorehabilitation. This concern is of course legitimate—it would be regrettable if the provision of neurorehabilitation led to the maintenance of the status quo where many prisons lack enrichment and/or where alternatives to imprisonment are not effectually pursued. However, it is not obvious that such an outcome would *inevitably* follow from neurorehabilitation use. It is, after all, also possible that wider use of neurorehabilitation to satisfy offenders’ rights against degeneration could draw attention to the deficiencies of our current carceral practices and precipitate greater momentum to exact change.^35^ Moreover, even if neurorehabilitation did delay reform of our detention conditions and punishment practices more generally, this would not undermine the argument that offenders have a right to neurorehabilitation when current practices can in fact be degenerative. It would be inhumane to deny offenders facing existing conditions a right to interventions that might make a meaningful difference to their criminal justice experience in order to incentivise penal reform. Withholding safe and effective neurorehabilitation solely for this reason would also arguably be an instance of using offenders as mere means to elicit such reform.

## The Right to Hope Justification

Let us next consider a positive justification for a right to rehabilitation—the argument that it is a derivative right of the right to genuine hope for renewed liberty, and/or (more generally) for a chance to turn one’s life around in the future.

This justification is increasingly prominent in the legal literature, though the literature varies in how it conceives of the right to hope. Sometimes the right to hope is purported to be about the hope for renewed liberty, with the emphasis placed on the need to preserve a right to hope in cases of indeterminate or life sentences. The German judiciary, for example, speaks of the importance of preserving offenders’ ‘hope of regaining [their] freedom’ and requires that the State ‘take all possible measures it can [reasonably] be expected to bear’ to give prisoners a ‘concrete and realistically attainable chance’ to achieve this.^36^ The ECtHR’s recent case law offers similar statements. Specifically, that prisoners, regardless of the severity of their wrongdoing, ‘retain the right to hope that … they may … atone for the wrongs they have committed’,^37^ and be given a ‘genuine and tangible’ ‘chance … to … regain their freedom’ through the provision of ‘real opportunity’ for rehabilitation.^38^ Other times the right to hope is presented as broader than that of hope for release, instead being viewed as a right to hope for release, reintegration and a prudentially better life post-punishment. Rotman, for instance, sees it as a right ‘to an opportunity to return to society with an improved chance of being a useful citizen and staying out of prison’.^39^ Brownlee likewise considers it to denote hope for ‘re-establish[ing] [oneself] in society’ and ‘lead[ing] a good life after punishment’—a right to hope for ‘a future after punishment [that is] *worth hoping for*’.^40^ In what follows then (and in the interests of remaining as inclusive as possible), I am understanding the right to hope in these broader terms—as the right to hope both for release and for a realistic chance of (re-)establishment in society, of staying out of prison and of leading a flourishing life in the future.

It is worth noting that this right to hope justification for a right to rehabilitation has four steps. The first involves advancing grounds for a right to genuine hope for liberation and reintegration, and the usual line of argument is that this right is required in order to afford offenders the respect their personhood or humanity demands. Denying offenders hope is argued to strike ‘at the core of human dignity’^41^ or (as Judge Power-Forde puts it) it is to deny offenders an ‘important and constitutive aspect of the human person’.^42^ And *all* offenders, so the reasoning goes, retain a right to hope for release and reintegration simply in virtue of their personhood, and regardless of the severity of their original crime(s) and their perceived dangerousness. The second step emphasises that this right to hope is, crucially, a right to *justified* or *realistic* hope. It is not that all offenders are entitled to be released and reintegrated. Nor is it that offenders have a right that a mere feeling of hope be generated within themselves. Rather, it is that offenders have a right to be afforded reasonable hope for such events to unfold—that any hopes they come to possess are reflective of a genuine (though not a certain) prospect of release and reintegration. This is what is key if we are to remain respectful of offenders as persons.^43^ The third step then involves recognising that justified hope for release and reintegration, in situations where this is dependent on the reduction of risk and/or changes in offenders’ dispositions, amounts to justified hope for the achievement of a rehabilitated state. The fourth step then derives a right to rehabilitative interventions from the above step, by insisting that these interventions are often necessary for achieving a rehabilitated state.

I anticipate some will take issue with one or more of these steps. I suspect some retributivists will maintain that when proportionate punishment for a given offence is a long or a life sentence, no right to hope for *earlier* liberation and reintegration exists. Some retributivists might also maintain that when a life sentence is understood literarily as a *sentence for life*, then there is no right to hope for liberation and reintegration at all.^44^ I further suspect that many will doubt that the provision of rehabilitative interventions is necessary for preserving hope. The argument might be that every offender *qua* rational agent possesses the capacity and potential to rehabilitate *themselves*, so why think a right to justified hope for rehabilitation requires that the State provide offenders with rehabilitation?

I do not intend to mount a thorough defence of the right to hope justification here. It is sufficient for my present purposes merely to note that the right to hope is widely recognised and supported, and as such provides a plausible ground on which to build my present argument for a right to neurorehabilitation.

However, in response to the retributivist objection, let me briefly reply that there is some level of inconsistency on the part of a retributivist who holds that punishment is owed to an offender if we are to respect them as persons or autonomous moral agents, but that hope (and recognition of the capacity for change) is *not* also owed to them for reasons of respect. As Brownlee suggests, evincing respect to persons plausibly entails not ‘giv[ing] up on’ them, recognising that even the most serious offenders (in the words of Avishai Margalit) ‘are worthy of basic human respect because of the possibility that … if given the opportunity, [they] may live the rest of their lives in a worthwhile manner’.^45^ The retributivist, then, is arguably guilty of respecting offenders on the one hand and disrespecting them on the other, if they maintain that punishment but not hope is deserved in the case of an offender serving a long or life sentence. Moreover, I do not think the retributivist need worry that (early) release, if achieved, would undermine proportionality. Indeed, the retributivist assumption that proportionate sentencing is *solely* determinable by desert assessments at the point of conviction is, in my view, one of the most implausible aspects of—but also something inessential to—retributivism. Intuitively, desert seems to be more appropriately understood as a dynamic concept.^46^ It is intuitive to judge that a person might deserve a certain amount of punishment for their wrongdoing at the point at which culpability is established but to later judge that this same amount of punishment is no longer deserved because that individual has (while receiving punishment) repented, reformed or endeavoured to make amends for their wrongful conduct. Acknowledging and preserving offenders’ right to hope thus need not undermine proportionality if we come to the discussion with an understanding of desert that is the more intuitive.^47^

The retributivist might persist, however, and claim that, even if desert *is* a dynamic concept, offenders serving ‘whole-life’ sentences ought not be *furnished* with a means to bring about their release. If these offenders rehabilitate themselves and a life sentence no longer appears appropriate or proportionate, the retributivist might allow that there should be a mechanism for their sentences to be reconsidered and reintegration facilitated, and concede that offenders should not be deprived of the hope of this possibility. But they might argue that those serving life sentences ought not have their prospect of release *increased* through rehabilitative intervention: that desert considerations dictate that we do not increase this prospect, even if respecting offenders entails that we not obliterate it altogether. Here, however, I simply disagree. I maintain, along with the abovementioned authors, that respecting offenders requires preserving justified hope insofar as it is possible, given the demands of justice. And while desert considerations might dictate that the prospect of release is justifiably *lower* or *more distant* for offenders serving whole-life sentences, I do not believe desert demands that the State prevent all and any improvement to this prospect by withholding rehabilitative assistance from offenders desirous of availing of it. There is, moreover, always the possibility that an offender’s life sentence could be overturned due to, for example, a procedural error. Thus, even if desert *does* demand that we refrain from improving life-sentenced offenders’ prospects of release, the right to hope still requires that such individuals be prepared for reintegration in the event that their sentence is overturned. And such preparation would involve the provision of rehabilitative assistance.

This brings us to the objection that the right to justified hope need not entail an entitlement to State-provided rehabilitation because offenders have the capacity to rehabilitate themselves. Here again, I disagree: I dispute that capacity for rehabilitation precludes the right to be assisted in its exercise. To be sure, all rational and autonomous agents are capable of re-evaluating their past conduct and subsequently choosing to lead a law-abiding life in the future. But it is overly demanding to expect people to rehabilitate themselves without support. The right to hope, after all, is surely most plausibly understood as a right to hope for rehabilitation, release and reintegration at *acceptable costs*, in terms of effort, to oneself—not a right to hope for rehabilitation through gargantuan effort. And there are many occasions where the absence of external support in facilitating rehabilitation would make the process a gargantuan task. It would, for instance, be extremely difficult for the offender whose wrongdoing stems in part from compulsive influences to attempt to curb these influences without support. It would also be very difficult for many offenders to successfully re-establish themselves in society *sans* assistance in improving their employability and job prospects. Thus, while the right to hope for rehabilitation and liberation might be contingent on one submitting to the demands of punitive or restorative justice, the right to justified hope for these outcomes at an acceptable cost to oneself requires that the State provide interventions and services to bring their achievement within easier reach.

My aim here, however, is to argue that the right-to-hope line of reasoning also supports a right to neurorehabilitation—that providing neurorehabilitation is sometimes necessary for offenders to be justified in hoping for reform, release and reintegration at non-gargantuan cost to themselves. Consider, for instance, the following illustrative cases.

***Adam***: Adam has been convicted of a violent assault and is serving a fixed-term prison sentence. His crime is appropriately characterised as an instance of impulsive aggression. It was not planned but rather ensued from Adam’s experiencing an extremely negative emotional reaction to a provocative stimulus.^48^ Adam has voluntarily partaken in various behavioural interventions aimed at ameliorating his impulsive and aggressive tendencies but they continue to be ineffective. He anticipates he will not be successful in controlling his aggressive reactions following release from prison.
***Denis***: Denis has been convicted of a sexual offence involving a 15-year-old child and is serving an indeterminate prison sentence. He does not meet the diagnostic criteria for paedophilia given the victim’s age.^49^ However, he experiences recurrent, sexual thoughts involving minors and has a history of previous sexual abuse of a minor. He has participated in a cognitive-behavioural/relapse prevention programme but still finds it difficult to control these thoughts. He anticipates that he will be unable to resist acting on his inappropriate urges if and when he is released in the future.
***Larry***: Larry has been convicted of murder and is serving a life sentence. He evinces little empathy for the victim of his crime and has difficulty empathising with the affective experience of others more generally. He has undergone substantial empathy and mindfulness training, but still demonstrates a very low level of empathic responsiveness to the pain and suffering of others. There are currently no plans to review his sentence.

The above three cases exhibit several differences but share, I think, one common feature. In each case, the respective offender lacks any real hope for rehabilitation and reintegration despite having had access to conventional rehabilitative interventions. In the latter two cases, moreover, justified hope for release is also presumably remote. I contend that in situations where conventional rehabilitative approaches prove (or can be expected to prove) ineffective in maintaining hope when used in isolation; and neurorehabilitation (in combination with these approaches) promises to be the more effective treatment package;^50^ then offenders have a right to avail of the latter provided the overall set of interventions remains affordable. On this reasoning then, the impulsively violent Alex who proves refractory to behavioural therapies has a right to pharmacotherapy to help curb his impulsive tendencies, as and when such therapy is available and affordable.^51^ Denis similarly has a right to anti-androgenic drugs or other agents which reduce sexual drive.^52^ And Larry has a right to avail of interventions that might enhance his ability to empathise with others, if and when such interventions exist.^53^

Someone might point out, however, that the right to hope justification only requires that the State provide interventions which offer the *hope* of rehabilitative success. It does not require that the State ‘fix’ things neurobiologically in individual cases where established (oft-successful) measures have proven ineffective.

Yet, neurorehabilitation (at least in the kinds of cases mentioned above) is not a biological ‘fix’.^54^ Attenuated aggression, reduced libido or enhanced empathy do not in-and-of-themselves lead to reformation and desistance from crime. Offenders still have to make the decision to desist. Just as caffeine helps facilitate concentration while leaving decisions about how to direct this newly focused state up to the individual consumer; the abovementioned neurointerventions promise to enhance (or reduce) certain tendencies or powers that plausibly will help facilitate rehabilitation and desistance if the offender is, in fact, *willing* to desist. Objections pertaining to the absence of a State duty to ‘fix’ are thus immaterial to my present line of argument.

The critic might instead argue that providing neurorehabilitation is inconsistent with the rationale underpinning the right to hope justification. We provide rehabilitation and recognise the right to hope out of respect for persons’ agential capacities—specifically, their rational and autonomous (RA) moral agency. Yet, neurorehabilitation, so the argument might go, is a paradigmatic instance of *disrespecting* a person’s RA moral agency, because it implies that genuine hope for rehabilitation is impossible in the absence of direct brain intervention.

Yet, this objection rests on an overly demanding conception of what RA moral agency involves. By this I mean, it erroneously restricts attributions of RA moral agency to those who alter their dispositions *exclusively* via rational means. I think we regularly attribute RA agency to people in other contexts when change transpires partly through *arational* means. We consider the writer who produces a successful novel to be exercising his RA agency in writing and garnering novelistic success, even if not a word of the novel could have been written without the author imbibing coffee or some other neurostimulant. We likewise consider the smoker who utilises nicotine patches or hypnotherapy to be exercising his RA agency in combating his addiction, regardless of whether his success ultimately depends on his use of these tools. And looking to the world of fiction, we deem Ebeneezer Scrooge to be a moral agent, capable of realising his past moral failures and acting differently henceforth, notwithstanding the fact that such a change of heart ultimately requires the arational influence of an epiphanic dream involving four ghosts.^55^ Returning to the neurorehabilitative context, then, I think the delivery of neurorehabilitation need not imply a negative judgement about offenders’ agential capacities. The candidate neurocorrectives mentioned above, after all, presume or rely upon their recipients’ capacity to reflect, form and pursue a conception of the good if they are to be effective. Interventions geared toward removing distracting impulses or attenuating aggression, for instance, assume those targeted have the requisite cognitive abilities for discerning and pursuing the good once relieved of these obstructive influences. Interventions to enhance empathy likewise assume the individual targeted has the capacity to choose and pursue a pro-social life once the experiencing of empathy is elicited. Neurorehabilitation, in these instances, thus *presumes* capacity. Why, after all, would we deliver *any* of these interventions if we judged offenders incapable of discerning and pursuing the good such that they would inevitably engage in further crime in any case?

The critic might reply that I am missing the point. It is precisely the implication that the offender cannot alter their dispositions exclusively through rational means that is disrespectful. It is *this* that sits oddly with a right to hope justification premised on respecting offenders as persons. Treating offenders as moral equals, so the reply might go, requires that we refrain from affording them differential treatment on the basis of their practical abilities.^56^ It requires that we assume all offenders—regardless of evidence to the contrary—are suitably responsive to rationality-engaging interventions and/or will be able to rehabilitate themselves exclusively through rational effort.

Here I think we have to ask ourselves which is the more disrespectful course of action. To assume an offender is equipped with all the internal resources necessary for achieving rehabilitation and desistance and to withhold potentially helpful neurointerventions even if this means continued detention. Or to recognise that the offender is not so equipped and provide, alongside rational engagement, an intervention which (temporarily) bypasses the offender’s rationality in order to facilitate their rehabilitation. I am inclined to think the former is more disrespectful. It might remain respectful of the offender as a RA agent (though even here, some have argued preventative detention is itself disrespectful of persons as RA agents).^57^ But it wholly disrespects the offender as a *social* agent for whom sustained social connection, and/or the hope of living within society, is a ‘constitutive part of a minimally decent human life’.^58^ Admittedly, it is commonplace (at least in philosophical circles) to consider RA agency as *the* feature of persons that merits our respect. But I have the intuition that respect is *also* due to people in virtue of their capacity to form sustained emotional attachments to others—in other words, their capacity to form relationships. And I think it would be *more* disrespectful of the State to deny an offender all hope of preserving, rekindling or building such relationships in the future by denying them neurorehabilitation, than to (arguably) express the message that their RA agency is somehow defective. I thus stand by my assertion that offenders are entitled to neurorehabilitation when this is necessary for preserving hope.

Before moving on to a final argument for offenders’ right to neurorehabilitation, let me briefly address whether this right-to-hope line of reasoning is really about rehabilitation, neuro or otherwise. Surely, someone might point out, if hope for establishment within society and of staying out of prison is a right of *persons*, then it applies equally to offenders and non-offenders. And if Adam, Denis or Larry came to the State *in advance* of offending (concerned about their abilities to refrain from criminal activity) then they too would have a right to neurointervention if it were necessary to preserve hope, and as and when it would be affordable.^59^

I agree. I accept that the right to hope for a non-carceral existence and establishment within society (and the right for assistance in securing this) is not limited to those who have offended. I realise, moreover, that the existence of distributive injustice means this right is frequently being infringed in the population more generally, and this is something that also warrants redress. But I do not think these observations undermine the claim that a right to neuro*rehabilitation* is a derivative right of the right to hope. Nor do I believe they imply that my argument here is not really about (neuro)rehabilitation. For one thing, it need not be the case that all possible grounds for offenders’ right to (neuro)rehabilitation must pertain uniquely to offenders. We can allow that *all* individuals have a right to assistance in maintaining a crime-free, socially integrated and well-functioning life (as and when this can be done at reasonable cost to the State); while still acknowledging that offenders specifically have a right to the means to sustain hope for the prerequisite—that they be released from the State’s grip. Imprisoned (or otherwise socially excluded) offenders, after all, have had their hopes for the above states-of-affairs *diminished even further* than they might have been prior to conviction. They not only are thwarted by lack of social capital and/or dispositional challenges or limitations, but also by the fact that the State is actively (albeit justifiably) preventing their pursuit of these states-of-affairs via its exclusionary mode of punishment. The right-to-hope line of reasoning thus supports the thought that the State is obliged to make available and offer (neuro)rehabilitation to offenders in order to enhance their hope for ‘in-society living’. But *offenders and non-offenders alike* are entitled to hope for more than this—and this obliges the State to provide both rehabilitative and ‘habilitative’ assistance insofar as it is feasible, and to work towards securing distributive justice.

## The Redressing Systemic Injustice Justification

This brings us nicely to a third argument supporting offenders’ right to rehabilitation—the duty to redress structural injustices, specifically, those injustices that have causally contributed to the offenders’ criminal activity. The thought here is that States are obliged, as a matter of justice, to compensate offenders for past State failures in addressing the root, socio-economic causes of crime, and rehabilitation is one key route to providing such recompense.

An obvious quibble at this point might be that the duty to combat structural injustice again does not ground a right to *rehabilitation*. It grounds a right to have the structural correlates of crime (e.g. poverty, concentrated disadvantage) addressed and requires that States afford offenders the rights that *they should have fulfilled anyway,* such as the right to education, healthcare, work and protection against unemployment.^60^ The mere fact that offenders are involved with the institutions of criminal justice necessitates these institutions are responsible for providing access to education, healthcare and the like. But it does not follow that offenders are entitled to anything *more*, following the commission of their crimes, than they should have received anyway. It is thus not so much ‘compensation’ and ‘a right to rehabilitation’ that we are talking about, but instead a simple entitlement to those basic human rights that are necessary for conferring equality of opportunity.

Again, I accept that a duty to combat structural injustice applies equally to offending and non-offending populations. I also acknowledge that much of what this duty requires is to respect, protect and fulfil people’s basic rights. However, I think that in situations where the State is causally responsible for experienced injustice, the act of tackling it is appropriately described as compensation.^61^ Moreover, those who have been victims of structural injustice *and* have committed crimes and been punished as a result, have had the structural injustice *compounded.* As such, some further recompense is plausibly required in these instances. We could, of course, also correct for this compounding injustice by punishing victims of structural injustice *less severely*. But when we have not done this (i.e. when structural injustice has not been accounted for in sentencing but has causally contributed to the offence) then States have a duty to compensate for this compounding injustice as well as the initial injustice. And rehabilitation is an obvious and appropriate mode of redress here, albeit that it is often coextensive with measures aimed at fulfilling people’s basic rights.

Yet, how might this justification extend to support a right to *neuro*rehabilitation? Surely (someone might suggest) this line of argument supports *psychosocial* and *social* interventions specifically? If State (and criminal justice) practices have helped to create a *social* and *familial* environment that is criminogenic, then surely the State’s reparative obligations comprise providing social support to families and communities, creating greater employment and reducing the socio-political harms occasioned by a criminal record, and providing mentoring interventions to the offender vulnerable to these external criminogenic influences. Problems with social origins, so the argument might go, warrant social solutions.

I concede that the vast portion of the State’s warranted response to State-perpetuated injustices ought to be so directed. However, I think it is a mistake to hold that offenders are *only* entitled to social forms of redress. For one thing, it is not obvious that social problems are *always* best remedied with social solutions in isolation. We might agree, for instance, that our current ecological crisis primarily requires radical changes in social priorities along with improved machinery to enforce these priorities; but we might also allow a place for biological interventions (e.g. soil carbon sequestration or afforestation) in mitigating the problem of climate change.^62^ More importantly, however, insisting on the exclusive use of social solutions in the structural injustice case is to ignore the extensive interplay of the environment and biology and the plasticity of the brain in response to environmental influence. Mounting neuroscientific evidence indicates, after all, that childhood poverty and its associated disadvantages are linked to damaged physiological stress regulatory mechanisms and alterations in brain development^63^—changes which appear, in turn, to contribute to a greater likelihood of developing low levels of self-control.^64^ This is not to say that poverty, disadvantage and/or childhood neglect *reliably* lead to these outcomes. Nor is it to say that low levels of self-control are *exclusively* found in individuals who have experienced poverty and disadvantage. Nevertheless, exposure to chronic stress and/or compromised caregiving during childhood—as might plausibly occur when families are experiencing financial, residential or other uncertainties—can be associated with changes in the functioning of the neural networks that underlie executive functions and self-regulation. And given that poor self-control is strongly associated with delinquency and criminal behaviour *and* that our knowledge of the biological substrates of self-control is ever increasing, neurorehabilitation might soon be part of the most effective approach toward ameliorating a problem that is social in origin but nevertheless amenable to biological intervention. I thus maintain that States will have a duty to provide it as and when it is safe and affordable to do so.

Two concerns might be voiced at this juncture. The first is that justifying neurorehabilitation for reasons of reparation risks ‘essentialising and homogenising poor people’—licensing differential treatment of offenders with low socio-economic status on the grounds that they are ‘neurobiologically impoverished’.^65^ The second holds that the disincentivising impact the availability of neurorehabilitation might have on State efforts to rectify structural injustice give us good reason *not* to invoke neurorehabilitation as a form of recompense to which offenders are entitled.

Yet, neither of these concerns undermine the claim that a right to neurorehabilitation can be at least *partly* grounded in the State’s duty to compensate for structural injustices. As to the first concern, this worry only arises if offenders’ right to neurorehabilitation were to be solely grounded in a State duty to compensate. We might risk essentialising and homogenising poor people if we were to hold that *only* victims of structural injustice were entitled to neurorehabilitation. But as already indicated, the right to neurorehabilitation can (and should) also be grounded in offenders’ right to hope and their right against degeneration through punishment—justifications which are open to all offenders regardless of socio-economic status. The State’s duty to compensate for injustice thus merely provides an *additional* justification for certain subpopulations of offenders but it is by no means the sole justification.

As to the second concern, I again dispute that risking disincentivising other important goals should lead us to deny offenders the right to neurorehabilitation. I concede it would be regrettable if neurorehabilitation were to distract attention away from the pursuit of much needed social reform. But this is an argument for the importance of continued advocacy with respect to these endeavours rather than for denying offenders’ neurorehabilitation. Moreover, I doubt that recognising a right to neurorehabilitation—if this right were at least partly grounded in a duty to redress structural injustices—would undermine the need to correct these injustices. If anything, it should draw attention to the multilevel impact of social and intergenerational injustice on individuals and hence reinforce the call to prevent such injustice going forward.

## Two Further Challenges

Thus far, I have detailed three justifications for a right to rehabilitation and argued these extend to support a right to neurorehabilitation. I have also anticipated and addressed some objections my argument might invite.

I now turn to briefly examine two further challenges that might be levelled at my argument—concerns about medicalisation and the ‘badness’ of the neurorehabilitative option.

### A Bad Option to Have

It might be objected that neurorehabilitation is a prudentially bad option for offenders to have, to the extent that it could impede offenders’ efforts to rehabilitate themselves and be detrimental to their self-esteem in cases where an offender would have preferred to strive for reform *sans* neurointervention. In other words that, in the presence of a neurorehabilitative offer, offenders might feel obliged to submit to neurorehabilitation even when they would prefer not to, and their resultant failure to ‘self-rehabilitate’ could negatively impact upon their sense of self-worth.

I realise that offering neurorehabilitation to offenders could have this undesirable consequence. However, I do not think it is an argument against recognising neurorehabilitation as a right. The objection draws attention to the importance of respecting, and affording scope for, offenders’ own reformative efforts within our criminal justice practices. It also indicates a need to counteract any negative impact of neurorehabilitation upon offender self-esteem, and this amounts to a call for the provision of further psychotherapeutic supports to recipients when appropriate. But it does not undermine the argument that offenders are entitled to avail of neurorehabilitation if and when conventional measures prove (or can be expected to prove) unfruitful. We elsewhere recognise some other rights that might be vulnerable to the same kind of objection. For instance, we (typically) recognise people’s right to adequate housing,^66^ often providing State-supported housing for those struggling to afford a place to live, even if availing of this support might be detrimental to some recipients’ self-esteem. We similarly recognise that people have a right to social security, and States often provide income security and support to those who need it—notwithstanding that receipt of such support (and hence the exercising of one’s right) might negatively affect a person’s sense of self-worth. Concerns about the ‘badness’ or otherwise of the neurorehabilitative option thus do not speak against the recognition of neurorehabilitation as a right. Instead, it simply highlights the need both for State care in its delivery and for adequate provision of alternative supports to those striving to rehabilitate themselves without neurorehabilitation.

### Medicalising Crime

My argument might also invoke the charge of over-medicalisation—that in granting offenders the right to neurorehabilitation, we unduly medicalise crime, painting an erroneous picture of a problem that is primarily social in origin and reverting criminological discourse to the best-forgotten pure biological theories of past.^67^

I accept that any arguments which promote the use of neurorehabilitation necessarily *medicalise* crime in making at least some instances of criminal behaviour a legitimate target for biomedical intervention. I dispute, however, that neurorehabilitation—and hence my argument for a right to neurorehabilitation—is an instance of *over*-medicalisation.

Just to be clear, my intention is not to downplay the importance and primacy of social factors and structures in contributing to the problem of crime. Nor is it to advance a deterministic picture of crime, either biological or social. Instead, I am simply coming from a perspective that considers crime to be a *biopsychosocially* embedded choice. That is, a choice that is influenced by socio-environmental, psychological and biological factors. And from this perspective, acknowledging a right to neurorehabilitation is *not* to unduly medicalise. It is instead to recognise that crime is the outcome of multiple, interrelating influences and to insist that offenders are entitled to have these multiple influences addressed through a multiplicity of means when such an approach can be expected to be effective. Just as we would consider a rehabilitative approach which *solely* focused on individuals’ psychological and biological characteristics to be misjudged and inappropriate, so too, I maintain, is an approach that ignores either of these sets of factors. Including neurorehabilitation within the purview of offenders’ entitlements, then, is an instance of appropriate medicalisation in my view, and not something to be condemned or regretted.

## Conclusion

To conclude, I have argued that considerations which support a moral right to rehabilitation extend to support a moral right to neurorehabilitation. I argued that offenders’ right to neurorehabilitation can be grounded in (at least) three ways: as a countermeasure to the degenerating impact of punishment; as a derivative right of the right to hope; and as recompense for structural injustice. I anticipated several objections that my argument might invite, including the charge of over-medicalisation and the worry that neurorehabilitation is a bad option for offenders to have. However, I maintained that none of these concerns undermine the central claim being advanced in this paper: that offenders have a moral right to neurorehabilitation when it would be part of the most effective package for facilitating rehabilitation and can be carried out at reasonable cost.

## Footnotes

1 Henceforth, offenders for short.

2 See Thomas Douglas and David Birks, ‘Introduction’, in *Treatment for Crime*, ed. David Birks and Thomas Douglas (Oxford: Oxford University Press, 2018) for this definition.

3 For an overview of the anti-libidinal agents used see Florence Thibaut et al., ‘The World Federation of Societies of Biological Psychiatry (WFSBP) Guidelines for the Biological Treatment of Paraphilias’, *The World Journal of Biological Psychiatry* 11, no. 4 (2010): 604–55.

4 Andrej Kastelic, Jörg Pont, and Heino Stoever, *Opioid Substitution Treatment in Custodial Settings* (Olderburg: BIS Verlag, 2009), http://www.ohrn.nhs.uk/resource/policy/OpioidSubs.pdf.

5 See Christopher Chew, Thomas Douglas, and Nadira S. Faber, ‘Biological Interventions for Crime Prevention’, in *Treatment for Crime*, ed. David Birks and Thomas Douglas (Oxford: Oxford University Press, 2018), 11–43, for an overview.

6 See Thomas Douglas, ‘Criminal Rehabilitation Through Medical Intervention: Moral Liability and the Right to Bodily Integrity’, *The Journal of Ethics* 18, no. 2 (1 June 2014): 101–22; Jan Christoph Bublitz, ‘“The Soul Is the Prison of the Body”: Mandatory Moral Enhancement, Punishment, and Rights Against Neurorehabilitation’, in *Treatment for Crime*, ed. David Birks and Thomas Douglas (Oxford: Oxford University Press, 2018), 289–320; and Elizabeth Shaw, ‘The Right to Bodily Integrity and the Rehabilitation of Offenders Through Medical Interventions: A Reply to Thomas Douglas’, *Neuroethics* 12, no. 1 (1 April 2019): 97–106, for representative discussion.

7 For example, the fictitious use of aversion therapy in Anthony Burgess’s *A Clockwork Orange*, Penguin Classics (London: Penguin, [1962] 2000). Also, the real-life example of the Connecticut START programme which sought to instigate behavioural change in child sex offenders by pairing the presentation of nude images of children with electric shocks, see Jean Hampton, ‘The Moral Education Theory of Punishment’, *Philosophy and Public Affairs* 13, no. 3 (1984): 222.

8 See Chew, Douglas, and Faber, ‘Biological Interventions for Crime Prevention’ for some examples.

9 HM Prison & Probation Service and Annual Report and Accounts 2019–20, *Costs per Place and Costs per Prisoner by Individual Prison* (Ministry of Justice UK, 2020), https://assets.publishing.service.gov.uk/government/uploads/system/uploads/attachment_data/file/929417/costs-prison-place-costs-prisoner-2019-2020-summary.pdf; Jeanne Hirschberger, ‘“Imprisonment Is Expensive”—Breaking down the Costs and Impacts Globally’, Penal Reform International, *Global Prison Trends 2020* (blog), 24 July 2020, https://www.penalreform.org/blog/imprisonment-is-expensive-breaking-down-the-costs-and/.

10 *Murray v. The Netherlands (10511/10)* [2016] ECHR para. 104.

11 *Harakchiev and Tolumov v. Bulgaria (15018/11 61199/12)* [2014] ECHR para. 264.

12 *Life Imprisonment Case*, 21 June 1977, 45 BVerfGE 187.

13 International Covenant on Civil and Political Rights (adopted 16 December 1966, entered into force 23 March 1976) 999 UNTS 171 (ICCPR).

14 I say less prominent because whilst it is often argued that we ought to rehabilitate offenders to redress structural injustice, it is less commonly argued that this reason grounds a *right* to rehabilitation.

15 Bublitz, ‘“The Soul Is the Prison of the Body”’.

16 *James, Wells and Lee v. The United Kingdom* (*25119/09, 57715/09 and 57877/09)* [2012] ECHR para. 218, mentions opportunities to ‘undertake courses aimed at helping [offenders] … address their offending behaviour and the risks they pose’; *Barnes v. Government of Virgin Islands*, 415 F. Supp. 1218 (District Court, Virgin Islands, 1976) speaks of ‘basic educational programs’, ‘vocational training’, ‘recreation’, ‘meaningful jobs’; *Laaman v. Helgemoe*, 437 F. Supp. 269 (District of New Hampshire, 1977) adds ‘library access’ and ‘social visitation’; and *Mitchell v. Untreiner*, 421 F. Supp. 886 (Northern District of Florida 1976) mentions ‘group and individual counselling’.

17 United Nations Congress on the Prevention of Crime and the Treatment of Offenders. 1958. *Standard minimum rules for the treatment of prisoners and related recommendations.* New York: United Nations, Dept. of Economic and Social Affairs, rule 62.

18 Emily McTernan, ‘Those Who Forget the Past: An Ethical Challenge from the History of Treating Deviance’, in *Treatment for Crime*, ed. David Birks and Thomas Douglas (Oxford: Oxford University Press, 2018), 287; Artemis Igoumenou, ‘The Use of Medication for the Treatment of Sex Offenders: Ethical Issues and Controversies’, in *Ethical Issues in Clinical Forensic Psychiatry*, ed. Artemis Igoumenou (Cham: Springer International Publishing, 2020), 51–83.

19 Timothy W. Kinlock et al., ‘A Study of Methadone Maintenance for Male Prisoners: 3-Month Postrelease Outcomes’, *Criminal Justice and Behavior* 35, no. 1 (2008): 34–47; Michael S. Gordon et al., ‘A Randomized Clinical Trial of Methadone Maintenance for Prisoners: Findings at 6 Months Post-Release’, *Addiction (Abingdon, England)* 103, no. 8 (August 2008): 1333–42.

20 Noa Krawczyk et al., ‘Only One In Twenty Justice-Referred Adults In Specialty Treatment For Opioid Use Receive Methadone Or Buprenorphine’, *Health Affairs* 36, no. 12 (2017): 2046–53; Amy Nunn et al., ‘Methadone and Buprenorphine Prescribing and Referral Practices in US Prison Systems: Results from a Nationwide Survey’, *Drug and Alcohol Dependence* 105, no. 1 (1 November 2009): 83–88.

21 Edgardo Rotman, ‘Do Criminal Offenders Have a Constitutional Right to Rehabilitation?’, *The Journal of Criminal Law and Criminology (1973-)* 77, no. 4 (1986): 1028.

22 National Research Council et al., ‘The Experience of Imprisonment’, in *The Growth of Incarceration in the United States: Exploring Causes and Consequences* (Washington, D.C., United States: National Academies Press, 2014), 174–201; Craig Haney, ‘Prison Effects in the Era of Mass Incarceration’, *The Prison Journal (Philadelphia, Pa.)*, 2012, 3288551244860; Carla Reeves, *Experiencing Imprisonment: Research on the Experience of Living and Working in Carceral Institutions* (London, United Kingdom: Taylor & Francis Group, 2015).

23 Christina Jose-Kampfner, ‘Coming to Terms with Existential Death: An Analysis of Women’s Adaptation to Life in Prison’, *Social Justice (San Francisco, Calif.)* 17, no. 2 (40) (1990): 110–25; R. J. Sapsford, ‘Life-sentence Prisoners: Psychological Changes During Sentence’, *British Journal of Criminology* 18, no. 2 (1978): 128–45.

24 Haney, ‘Prison Effects in the Era of Mass Incarceration’, 5.

25 Kristine Levan, *Prison Violence: Causes, Consequences, and Solutions*, Solving Social Problems (London [England]: Routledge, 2016).

26 J. Meijers et al. "Reduced Self-Control after 3 Months of Imprisonment; A Pilot Study." *Frontiers in Psychology* 9 (2018): 69.

27 Shelley Johnson Listwan et al., ‘The Pains of Imprisonment Revisited: The Impact of Strain on Inmate Recidivism’, *Justice Quarterly* 30, no. 1 (1 February 2013): 144–68; Corey M. Leidenfrost and Daniel Antonius, ‘Incarceration and Trauma: A Challenge for the Mental Health Care Delivery System’, in *Assessing Trauma in Forensic Contexts*, ed. Rafael Art. Javier, Elizabeth A. Owen, and Jemour A. Maddux (Cham: Springer International Publishing, 2020), 85–110; Mika’il DeVeaux, ‘The Trauma of the Incarceration Experience’, *Harvard Civil Rights-Civil Liberties Law Review* 48, no. 1 (2013): 277.

28 Ioan Durnescu, ‘Pains of Probation: Effective Practice and Human Rights’, *International Journal of Offender Therapy and Comparative Criminology* 55, no. 4 (1 June 2011): 530–45.

29 *Laaman v. Helgemoe* 437 F. Supp. 269 (District of New Hampshire, 1977) at 323.

30 Dennis S. Charney and Steven M. Southwick, eds., ‘What Is Resilience?’, in *Resilience: The Science of Mastering Life’s Greatest Challenges*, 2nd ed. (Cambridge: Cambridge University Press, 2018), 1–34.

31 Some of these agents have already entered early-stage clinical trials, see Allyson K. Friedman et al., ‘KCNQ Channel Openers Reverse Depressive Symptoms via an Active Resilience Mechanism’, *Nat Commun* 7, no. 1 (2016): 11671; Aaron Tan et al., ‘Effects of the KCNQ Channel Opener Ezogabine on Functional Connectivity of the Ventral Striatum and Clinical Symptoms in Patients with Major Depressive Disorder’, *Molecular Psychiatry* 25, no. 6 (June 2020): 1323–33.

32 Monique F. Crane et al., ‘Strengthening Resilience in Military Officer Cadets: A Group-Randomized Controlled Trial of Coping and Emotion Regulatory Self-Reflection Training’, *J Consult Clin Psychol* 87, no. 2 (2019): 125–40.

33 Both prior to and within the correctional setting, see Nancy Wolff and Jing Shi, ‘Childhood and Adult Trauma Experiences of Incarcerated Persons and Their Relationship to Adult Behavioral Health Problems and Treatment’, *International Journal of Environmental Research and Public Health* 9, no. 5 (May 2012): 1908–26; Craig Haney, ‘The Psychological Impact of Incarceration: Implications for Post-Prison Adjustment’ (Washington D.C.: Office of the Assistant Secretary of Planning and Evaluation, 23 November 2015), https://aspe.hhs.gov/basic-report/psychological-impact-incarceration-implications-post-prison-adjustment.

34 Durnescu, ‘Pains of Probation: Effective Practice and Human Rights’.

35 See Katrien Devolder, ‘Euthanasia for Detainees in Belgium’, *Camb Q Healthc Ethics* 25, no. 3 (2016): 384–94, for a related example where a Belgian prisoner’s request for euthanasia served as a trigger for widespread public debate about Belgium’s detention conditions.

36 *Life Imprisonment Case*, 21 June 1977, 45 BverfGE 187.

37 Concurring opinion of Judge Power-Forde in the case of *Vinter and others v. United Kingdom* (*66069/09, 130/10 and 3896/10*) [2013] ECHR.

38 *Harakchiev and Tolumov v. Bulgaria (15018/11 and 61199/12)* [2014] ECHR para. 264.

39 Rotman, ‘Do Criminal Offenders Have a Constitutional Right to Rehabilitation?’: 1027.

40 Emphasis added. See Kimberley Brownlee, ‘Punishment and Precious Emotions: A Hope Standard for Punishment’, *Oxford Journal of Legal Studies* 41, no. 3 (1 September 2021): 602–03.

41 *Life Imprisonment Case*, 21 June 1977, 45 BverfGE 187.

42 Concurring opinion of Judge Power-Forde in the case of *Vinter and others v. United Kingdom* (*66069/09, 130/10 and 3896/10*) [2013] ECHR.

43 Brownlee, ‘Punishment and Precious Emotions’, 595.

44 I acknowledge that few penal theorists consider these sentences to be proportionate for anything but the most extreme offenses (for example, genocide).

45 Avishai Margalit, *The Decent Society* (Cambridge, Mass: Harvard University Press, 1996), cited in Kimberley Brownlee, ‘Punishment and Precious Emotions: A Hope Standard for Punishment’, *Oxford Journal of Legal Studies* 41, no. 3 (1 September 2021): 605.

46 See Netanel Dagan and Julian V. Roberts, ‘Retributivism, Penal Censure, and Life Imprisonment without Parole’, *Criminal Justice Ethics* 38, no. 1 (2 January 2019): 1–18, for a persuasive argument to this effect.

47 For discussion of some of the theoretical challenges a dynamic desert model might create see Jesper Ryberg, ‘Retributivism and the Dynamic Desert Model: Three Challenges to Dagan and Roberts’, *Criminal Justice Ethics* 40, no. 1 (2021): 56–67.

48 For a distinction between impulsive and instrumental aggression see Robert J. R. Blair, ‘The Neurobiology of Impulsive Aggression’, *Journal of Child and Adolescent Psychopharmacology* 26, no. 1 (February 2016): 4–9.

49 The Diagnostic and Statistical Manual of Mental Disorders, Fifth Edition (DSM-5) specifies that a diagnosis of paedophilia requires, amongst other things, the experiencing of ‘intense sexually arousing fantasies or urges involving sexual activity with *prepubescent* children’, see *Diagnostic and Statistical Manual of Mental Disorders: DSM-5*, Fifth, DSM-5 (Washington, DC: American Psychiatric Publishing, 2013).

50 I say ‘in combination’ because it is conceivable that pharmacological/brain stimulation therapies *without* associated psychotherapy or counselling might give rise to negative and counterproductive side-effects.

51 Tony Butler et al., ‘Sertraline Hydrochloride for Reducing Impulsive Behaviour in Male, Repeat-Violent Offenders (ReINVEST): Protocol for a Phase IV, Double-Blind, Placebo-Controlled, Randomised Clinical Trial’, *BMJ Open* 11, no. 9 (1 September 2021); Dongju Seo, Christopher J. Patrick, and Patrick J. Kennealy, ‘Role of Serotonin and Dopamine System Interactions in the Neurobiology of Impulsive Aggression and Its Comorbidity with Other Clinical Disorders’, *Aggression and Violent Behavior* 13, no. 5 (October 2008): 383–95.

52 See Don Grubin, ‘The Pharmacological Treatment of Sex Offenders’, in *The Wiley Blackwell Handbook of Forensic Neuroscience*, ed. Anthony R. Beech et al. (John Wiley & Sons, Incorporated, 2018), 703–23 for an overview of the various agents that might be effective in this regard.

53 The research here is still highly speculative. However, there is some small-scale evidence to suggest that brain stimulation techniques and pharmaceutical interventions might be capable of enhancing empathy, see Sébastien Hétu, Vincent Taschereau-Dumouchel, and Philip L. Jackson, ‘Stimulating the Brain to Study Social Interactions and Empathy’, *Brain Stimul* 5, no. 2 (2012): 95–102; Molly J. Crockett et al., ‘Serotonin Selectively Influences Moral Judgment and Behavior through Effects on Harm Aversion’, *Proc Natl Acad Sci U S A*, From the Cover, 107, no. 40 (2010): 17433–38; and Jennifer A. Bartz et al., ‘Oxytocin Selectively Improves Empathic Accuracy’, *Psychol Sci* 21, no. 10 (2010): 1426–28.

54 I concede that a neuro-implant which sought to control an offender’s decision-making would provide such a ‘fix’ but these are not the kinds of neurointerventions we are talking about.

55 Charles Dickens, *A Christmas Carol and Other Christmas Books*, ed. Robert Douglas-Fairhurst, Oxford World’s Classics (Oxford: Oxford University Press, 2008).

56 Christopher Bennett, ‘Intrusive Intervention and Opacity Respect’, in *Treatment for Crime*, ed. David Birks and Thomas Douglas (Oxford University Press, 2018), 255–73.

57 Andrew von Hirsch, ‘Predication of Criminal Conduct and Preventive Confinement of Convicted Persons’, *Buffalo Law Review* 21 (1971): 717; Barbara D. Underwood, ‘Law and the Crystal Ball: Predicting Behavior with Statistical Inference and Individualized Judgment’, *The Yale Law Journal* 88, no. 7 (1979): 1408–48.

58 Kimberley Brownlee, ‘A Human Right Against Social Deprivation’, *The Philosophical Quarterly* 63, no. 251 (2013): 1.

59 I am grateful to an anonymous reviewer for raising this point.

60 United Nations. General Assembly, *Universal Declaration of Human Rights* (Lake Success, N.Y.: United Nations Department of Public Information, 1948).

61 Here, I set aside the problem of non-identity, Derek Parfit, *Reasons and Persons*, 2nd repr. with corrections of 1984 (Oxford: Clarendon Press, 1987), 351–80. But see Ori J. Herstein, ‘Historic Justice and the Non-Identity Problem: The Limitations of the Subsequent-Wrong Solution and towards a New Solution’, *Law and Philosophy* 27, no. 5 (2008): 505–31, for a possible solution.

62 W. Amelung et al., ‘Towards a Global-Scale Soil Climate Mitigation Strategy’, *Nature Communications* 11, no. 1 (27 October 2020): 5427; Jonathan C. Doelman et al., ‘Afforestation for Climate Change Mitigation: Potentials, Risks and Trade-Offs’, *Global Change Biology* 26, no. 3 (1 March 2020): 1576–91.

63 Gary W. Evans and Pilyoung Kim, ‘Childhood Poverty and Health: Cumulative Risk Exposure and Stress Dysregulation’, *Psychological Science* 18, no. 11 (2007): 953–57; Amanda R. Tarullo and Megan R. Gunnar, ‘Child Maltreatment and the Developing HPA Axis’, *Translational Topics in Behavioral Neuroendocrinology* 50, no. 4 (1 November 2006): 632–39; Jack P. Shonkoff, W. Thomas Boyce, and Bruce S. McEwen, ‘Neuroscience, Molecular Biology, and the Childhood Roots of Health Disparities: Building a New Framework for Health Promotion and Disease Prevention’, *JAMA* 301, no. 21 (3 June 2009): 2252–59.

64 Bruce D. Perry et al., ‘Childhood Trauma, the Neurobiology of Adaptation, and “Use-Dependent” Development of the Brain: How “States” Become “Traits”’, *Infant Mental Health Journal* 16, no. 4 (1 December 1995): 271–91; Matt Vogel and Maarten Van Ham, ‘Unpacking the Relationships between Impulsivity, Neighborhood Disadvantage, and Adolescent Violence: An Application of a Neighborhood-Based Group Decomposition’, *Journal of Youth and Adolescence* 47, no. 4 (1 April 2018): 859–71; Daniel Romer, ‘Adolescent Risk Taking, Impulsivity, and Brain Development: Implications for Prevention’, *Developmental Psychobiology* 52, no. 3 (April 2010): 263–76.

65 Victoria Pitts-Taylor, ‘Neurobiologically Poor? Brain Phenotypes, Inequality, and Biosocial Determinism’, *Science, Technology, & Human Values* 44, no. 4 (2019): 678.

66 I say ‘typically’ because some nations have not ratified the International Covenant on Economic, Social and Cultural Rights.

67 Cesare Lombroso, *Criminal Man*, trans. Mary Gibson and Nicole Hahn Rafter (Durham, NC: Duke University Press, 2006).

